# Recent Advances in Materials for Wearable Thermoelectric Generators and Biosensing Devices

**DOI:** 10.3390/ma15124315

**Published:** 2022-06-18

**Authors:** Maria Sattar, Woon-Hong Yeo

**Affiliations:** 1 George W. Woodruff School of Mechanical Engineering, Georgia Institute of Technology, Atlanta, GA 30332, USA; msattar6@gatech.edu; 2 IEN Center for Human-Centric Interfaces and Engineering, Institute for Electronics and Nanotechnology, Georgia Institute of Technology, Atlanta, GA 30332, USA; 3 Wallace H. Coulter Department of Biomedical Engineering, Parker H. Petit Institute for Bioengineering and Biosciences, Georgia Institute of Technology, Atlanta, GA 30332, USA; 4 Neural Engineering Center, Institute for Materials, and Institute for Robotics and Intelligent Machines, Georgia Institute of Technology, Atlanta, GA 30332, USA

**Keywords:** thermoelectric material, energy harvesting, nanogenerator, wearable device, self-powered system

## Abstract

Recently, self-powered health monitoring systems using a wearable thermoelectric generator (WTEG) have been rapidly developed since no battery is needed for continuous signal monitoring, and there is no need to worry about battery leakage. However, the existing materials and devices have limitations in rigid form factors and small-scale manufacturing. Moreover, the conventional bulky WTEG is not compatible with soft and deformable tissues, including human skins or internal organs. These limitations restrict the WTEG from stabilizing the thermoelectric gradient that is necessary to harvest the maximum body heat and generate valuable electrical energy. This paper summarizes recent advances in soft, flexible materials and device designs to overcome the existing challenges. Specifically, we discuss various organic and inorganic thermoelectric materials with their properties for manufacturing flexible devices. In addition, this review discusses energy budgets required for effective integration of WTEGs with wearable biomedical systems, which is the main contribution of this article compared to previous articles. Lastly, the key challenges of the existing WTEGs are discussed, followed by describing future perspectives for self-powered health monitoring systems.

## 1. Introduction

**Figure 1:**
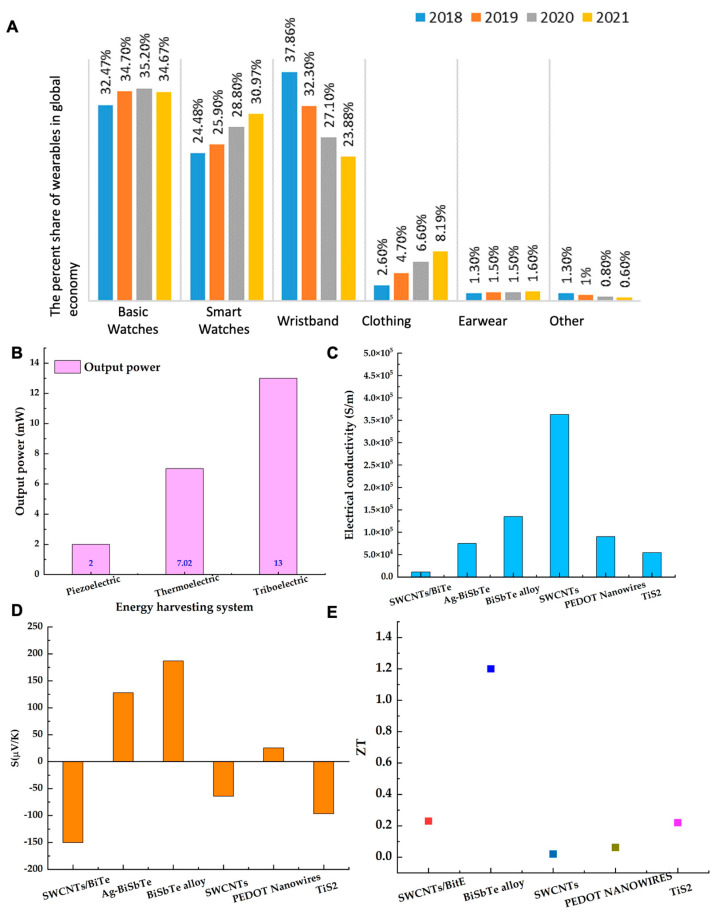
(**A**) The market share of wearable electronics in the global market between 2018 and 2021; reprinted from Ref. [2]. Copyright year (2020), Nozariasbmarz, A.; Collins, H.; Dsouza, K.; Polash, M.H.; Hosseini, M.; Hyland, M.; Liu, J.; Malhotra, A.; Ortiz, F.M.; Mohaddes, F.; et al. with permission from Elsevier; (**B**) The comparative analysis of the maximum reported output power generated by piezoelectric, thermoelectric, and triboelectric [9,10,21]. (**C**–**E**) The comparative analysis of electrical conductivities, Seebeck coefficient, and ZT of organic, inorganic, hybrid matrix, and polymer at room temperature for WTEG application, respectively [18,19,20,22,23]. (The ZT value of Ag-BiSbTe was not given, and it cannot be calculated because thermal conductivity of film was not reported in the article—[23]).

Self-powered health monitoring systems are employed mainly to track the health vitals of players during exercise and training and for the regular health monitoring of elders. These systems are usually powered by batteries charged either by a conventional power source or by wearable energy harvesting devices, i.e., ferroelectric, thermoelectric, triboelectric, and piezoelectric [1]. Currently, our lives have been dramatically changed with the emergence of these wearable health monitoring systems. As a result, the advent of the internet of things, “IoT,” has increased the energy demand for wearable devices [2]. Furthermore, the purchase of personalized clothing and smartwatches has increased from 2.6% to 8.19% and 24.48% to 30.97% in 2021, respectively, as shown in Figure 1A [2]. This increase in wearables’ adaptability has resulted in exploring freely and readily available renewable energy resources to meet the energy demand without carbon footprint [3,4]. Energy harvesting systems, such as ferroelectric, thermoelectric, triboelectric, and piezoelectric, harvest natural forms of energy, i.e., biomechanical, thermal, mechanical, and vibrational energies, to power the wearables, respectively [5,6,7].

A ferroelectric generator works on the principle of the direct piezoelectric effect and can be piezoelectric and pyroelectric at the same time. It uses spontaneous electric polarization, which can be actuated by a change in stress or temperature, resulting in the considerable potential to power a biosensing system [7]. A piezoelectric generator converts human biomechanical energy into electrical energy by using the piezoelectric effect and generates a voltage of up to 55 V when force is exerted by the human foot [5]. Such a high voltage generation increases the scope of piezoelectric to power biosensing devices. However, the abrupt output power at low frequency limits its real-time application to biosensors [8,9]. In addition, it requires an active state to harvest biomechanical energy. A triboelectric nanogenerator (TENG) harvests mechanical energy during surface charge transfer between two oppositely charged surfaces at a reasonable distance [10]. TENG has a wide area of applications and has successfully worked as a self-powered pressure sensor [11]. Using the Seebeck effect, a TEG harvests human body heat and converts it directly into electrical energy. In recent years, TEG has successfully powered various biosensors at a lab-scale [12]. The key advantage of these energy harvesting systems is their multifunctionality [11]. Along with energy harvesting, piezoelectric works to detect human motion as a strain sensor, TENG works as a pressure sensor, and TEG works as a temperature sensor. A comparative analysis of output power generated from TEG, TENG, and piezoelectric is given in Figure 1B, which shows that the thermoelectric system has a slightly lower output power compared to the triboelectric system. However, TEGs are capable of harvesting human body heat in any state, i.e., at rest or during work, without extra effort. Moreover, TEGs offer no-cost cooling to smart electronics, which is an added feature of this renewable energy harvesting system [13,14].

A thermoelectric generator (TEG) is a solid-state device built on a semiconducting material. It directly converts thermal energy into electrical energy by using the Seebeck effect. Recently, there has been considerable improvement in the thermoelectric properties, i.e., the electrical conductivity (σ), Seebeck coefficient (S), and figure of merit (ZT), of thermoelectric (TE) materials [13]. Highly sophisticated equipment is used to measure these TE properties. The four-point probe and laser flash method measure the electrical conductivity and the thermal conductivity of TEG, respectively [15,16]. The Seebeck coefficient is measured by dividing the difference in voltage at room temperature and required temperature by the difference in temperatures (S = −ΔV/ΔT). Commercial equipment, such as the ULVAC ZEM-3 and thin-film analyzer, is used to measure Seebeck coefficients and TE power [16,17]. Figure 1C–E show a comparative analysis of electrical conductivities, Seebeck coefficient, and ZT for frequently reported thermoelectric materials. It can be seen from Figure 1C that the electrical conductivity varies from 10^3^ to 10^5^ S/m for TE materials. A recent study reported an electrical conductivity of 3.63 × 10^5^ S/m for n-type SWCNT film, which is a huge development for TEs [18]. However, the Seebeck coefficient is higher for inorganic materials, i.e., ~187 µV/K for bulk BiTe alloy [19] and −96 µV/K for TiS_2_ [20], which directly increases the ZT as shown in Figure 1D,E. Considerable efforts are necessary to improve the Seebeck coefficient of SWCNTs and polymer materials. This is because the SWCNT-based films have a thickness of a few microns and exhibited a planar architecture that impacts the generation of voltage at both ends. In recent studies, the focus has been to address the challenges of these highly electrically conductive materials, and innovative designs are being investigated. In Section 4 and Section 5, we will discuss additional details and proposed future directions to address the aforementioned challenges.

**Figure 2:**
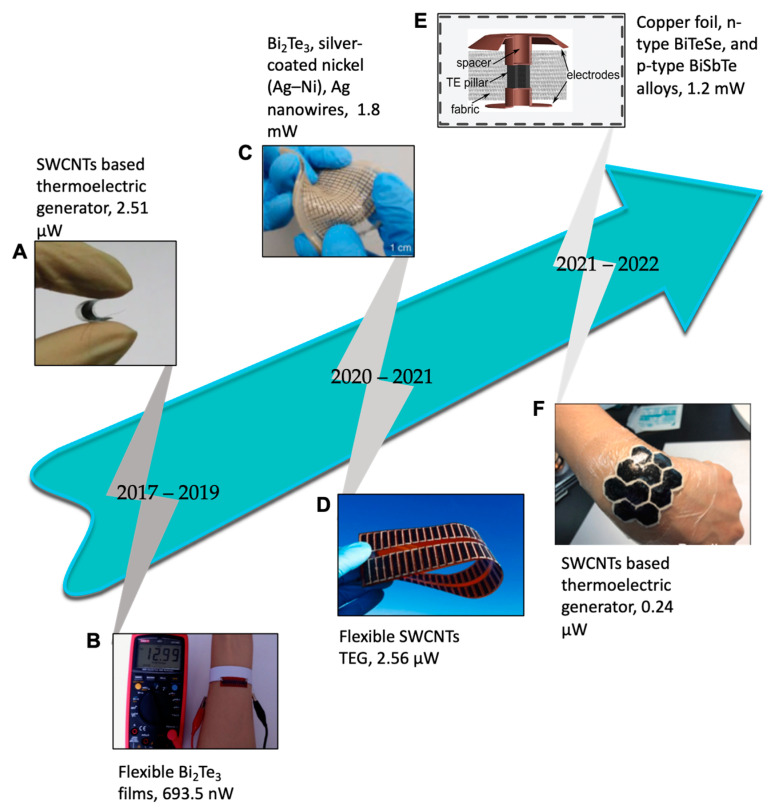
The advancement in the system architecture and the increase in power output of flexible TEGs within the last five years. (**A**) SWCNT-based TEG, reprinted from [18], copyright (2017), Zhou, W.; Fan, Q.; Zhang, Q.; Cai, L.; Li, K.; Gu, X.; Yang, F.; Zhang, N.; Wang, Y.; Liu, H.; et al. with permission from nature communications; (**B**) flexible Bi_2_Te_3_ film, reprinted from [15], copyright (2019), Kong, D.; Zhu, W.; Guo, Z.; Deng, Y. with permission from Elsevier; (**C**) Bi_2_Te_3_, silver-coated nickel (Ag–Ni), Ag nanowire-based TEG, reprinted from [21] copyright (2020), Lee, B.; Cho, H.; Park, K.T.; Kim, J.-S.; Park, M.; Kim, H.; Hong, Y.; Chung, S., with permission from Nature communications; (**D**) flexible SWCNTs TEG, reprinted from [24], copyright (2021), Mytafides, C.K.; Tzounis, L.; Karalis, G.; Formanek, P.; Paipetis, A.S. with permission from Elsevier; (**E**) copper foil, n-type BiTeSe, and p-type BiSbTe alloys, reprinted from [25]; (**F**) SWCNT-based e-skin TEG, reprinted from [26], copyright (2021), Kim, M.H.; Cho, C.H.; Kim, J.S.; Nam, T.U.; Kim, W.-S.; Lee, T.I.; Oh, J.Y. with permission from Elsevier.

Figure 2 shows the advancement in the design and soft packaging of WTEG for flexibility and stretchability [15,18,21,24,25,26]. It indicates considerable improvements in the thermoelectric performance of flexible TEGs [18,19,22,23]. It is noteworthy that soft packaging improved the strain tolerance from 3% to 20% for inorganic thermoelectric material-based flexible TEGs, which successfully generated thermoelectric power from ~693.5 nW (13 np pairs, ΔT = 24 K) to ~7.02 mW (220 np pairs, ΔT = 40 K) [15,21]. Although organic material-based TEG devices successfully generated ~39 µW (336 np pairs, ΔT = 50 K), however, these could only generate 2.6 µW at ΔT = 6 K for wearable applications, which is sufficient to power a low-power biosensor with a good strain tolerance [24]. Another parameter, magnetothermopower, to improve the thermoelectric performance of TEGs has been introduced. This parameter has a strong relationship with the Seebeck coefficient (S) in magnetic semiconducting material [27]. It is reported that an external magnetic field has a strong impact on the electrical conductivity, thermal conductivity, and Seebeck coefficient. In ferromagnetic semiconducting material, ferrons exist at the Curie temperature and fall quickly as the outside magnetic field changes at T > room temperature (RT). This fall of ferrons generates a large potential difference, which eventually improves the ZT of the TEG [28]. Further exploration of its impact on the performance of TEG is required for magnetic semiconducting TE materials. However, this review only focuses on nonmagnetic semiconducting materials.

Thermoelectric generator-powered wearable health monitoring sensors utilize body heat to monitor health vitals. TEG-powered biosensors require mechanical robustness, flexibility, and stretchability to overcome the strain effect during the working state. Despite the advances in biosensors, the integration of WTEGs with biosensors is difficult. Currently, the research focus of WTEGs has shifted to film-based WTEGs to achieve active integration. It is necessary for WTEGs to be in good conformity with the human body, which ensures the maximum heat conduction through interfacial layers and stabilizes the thermal gradient. A TEG-based biosensing system is comprised of six main components: (1) TEG, (2) integrated power management system (IPMS), (3) interconnects, (4) biosensor, (5) actuator, and (6) transmission system [12]. For powering the biosensing system, the WTEG must be well adhered to the human skin to generate enough voltage to not only derive the IPMS but also power the biosensor with a good strain tolerance [21]. Currently, the WTEGs built on TE legs have a low strain tolerance, hence the trends in the fabrication of WTEGs shifting to film-based TEGs to offer good interfacial strength and compatibility with biosensing devices [17,29,30,31]. A detailed discussion is provided in Section 7, Section 8 and Section 9. We formulated this review by first discussing the challenging aspects of thermoelectric materials for WTEG and their performance and suggesting measures to improve the TE properties. Then, the designs and packaging methods of WTEGs are reviewed and future recommendations are proposed. Lastly, energy system analysis is performed to effectively integrate the WTEG with a wearable health monitoring system by reducing the overload energy budget. Future directions are given for advancing the WTEGs.

## 2. Why Thermoelectrics?

**Figure 3:**
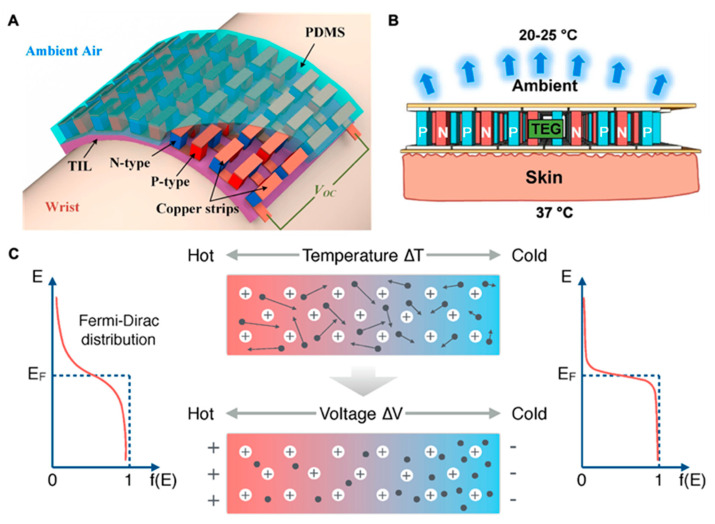
(**A**) A schematic of WTEG on a human wrist, reprinted from [45], copyright (2017), Wang, Y.; Shi, Y.; Mei, D.; Chen, Z, with permission from Elsevier; (**B**) the cross-sectional view of the thermoelectric process in WTEG on human skin, reprinted from [2], copyright(2019), Nozariasbmarz, A.; Collins, H.; Dsouza, K.; Polash, M.H.; Hosseini, M.; Hyland, M.; Liu, J.; Malhotra, A.; Ortiz, F.M.; Mohaddes, F, with permission from Elsevier; (**C**) the Seebeck effect in the WTEG, reprinted from [41], copyright (2020), Park, J.; Bhat, G.; Nk, A.; Geyik, C.S.; Ogras, U.Y.; Lee, H.G, with permission from MDPI.

In a recent annual energy outlook report of EIA USA, a sharp surge in the energy demand has been projected, and a 30% rise in energy consumption from 2020 to 2050 has been predicted [32,33]. This increase in energy consumption is somehow related to the emergence and adaptability of electronics, especially wearable electronics for personal use [34]. In recent years, this increase in energy demand has anticipated the search for new ways to power wearable electronics and the exploration of renewable energy resources, i.e., photovoltaics (PVs) and thermoelectric (TE), as power sources for wearable electronic devices. The energy generation from PVs and thermoelectrics has several advantages, i.e., a direct, immediate, and environmentally friendly power supply; however, energy harvesting from PVs has a few disadvantages. It is a fact that solar energy is only available in the daytime, which causes its limited availability and restricts its application to wearable technology, especially for active tracking and health monitoring devices in off-hours. Thermoelectric has the potential to power wearables without interruption during off-hours. A typical fabrication process for a WTEG includes four simple steps: 1—fabrication of bottom and top electrodes; 2—fabrication of p- and n-type legs; 3—placement of n- and p-type legs over electrodes and soldering them; and 4—soft packaging of the WTEG. It exhibits the advantage of converting human body heat into electrical energy at very low-temperature differences, i.e., ΔT = ~3 K, by using the Seebeck effect of thermoelectric material and generates electrical energy from a few nW to several mW [19,22,24,35] (Figure 3A). It is advantageous because the human thermoregulatory system continuously regulates the human body temperature to 37 °C (Figure 3B). However, its energy conversion efficiency has yet to improve [36,37,38]. A WTEG is a solid-state device made up of semiconducting material. It comprises n- and p-type pairs connected electrically in series and thermally in parallel. It converts thermal energy into electrical energy by using the Seebeck effect. The Seebeck effect generates a voltage potential between hot and cold sides when a temperature gradient ($\Delta T=T_{\mathrm{hot}}-T_{\mathrm{cold}}$) is established across the WTEG (Figure 3C) and measured in terms of the Seebeck coefficient (S=−ΔV/ΔT) [26,34,39,40,41]. In TE materials, the energy states of the electrons and holes establish the energy transfer inside the TE legs, which is measured by the Fermi–Dirac distribution. The Fermi–Dirac distribution was invented by Fermi and Dirac in 1926 to estimate the number of active electrons [42,43]. (The Reference [42] of the Fermi–Dirac distribution is the translated version of the original article written by Fermi, which is not found on the internet. This version was translated by the A. Zannoni Institute for Physical Science and Technology, University of Maryland at College Park, College Park, Maryland 20742 and the Electron and Optical Physics Division, National Institute of Standards and Technology, Gaithersburg, Maryland 20899). It is used to calculate the energy densities of the electrons responsible for current flow in the state of thermodynamic equilibrium. It determines the potential of similar electrons at different energy levels, which follows the Pauli exclusion principle in half spin (1/2) [43,44]. The average number of active electrons (fermions) is measured by Equation (1) [44]. When moving under a thermal gradient across the p- and n-type junction, these energetic electrons generate a voltage difference under the Seebeck effect [41]:

(1) ni=11+e(εi−μ)TkB

where *ε_i_* is the energy of the single-particle state, *μ* is the total chemical potential, T is the absolute temperature, and k_B_ is Boltzmann’s constant. The integration of these low-temperature operating devices with smart wearable technology will not only improve the functionality of wearable electronics, but it will also reduce the dependency on batteries. The power management system will be equipped with a voltage regulator that will regulate the voltage through supercapacitors and power the wearables directly or with a battery. Thus, without the need for a battery, the wearable device can maintain functions for health monitoring, which is the most significant advantage of TEGs over triboelectric, ferroelectric, and piezoelectric generators. It will eventually help to mitigate the environmental footprint of wearable electronics.

## 3. An Overview of Key Challenges in Developing WTEG

This section will discuss the key challenges associated with the TE materials, design of WTEGs, soft packaging, and integration with biosensors. Previously, the primary focus of TE research has been to improve the energy conversion efficiency of the TEG by introducing new materials and innovative design strategies. Recent studies insisted on developing lightweight, flexible, and highly conformal WTEGs. These emphasized the use of nanomaterials and the addition of a heat sink, which have proved to be effective measures to stabilize the thermoelectric gradient across TEGs in industrial applications [14,33,34]. For the manufacturing of flexible WTEGs, certain material challenges need to be addressed. For instance, inorganic materials have good TE properties in bulk; however, the fabrication of TE films from bulky materials is costly and time-consuming. Although CNTs offer manufacturing advantages over inorganic materials, their TE properties need to be optimized. Although soft packaging helps to improve the strain tolerance of WTEGs, it does not offer good conformity for TE leg-based WTEGs. Another challenge is that biosensors become flexible, soft, and miniature, which creates a design mismatch with the currently available WTEGs. Along with this, the manufacturing of TE legs is time-consuming and labor-intensive. Recent efforts have focused on introducing easy-to-fabricate processes for WTEGs. Furthermore, WTEG research lacks the energy budgeting of the entire system. Jaehyun Park et al. developed an analytical model to perform the energy per optimization of wearable IoT devices by using the PV module and maximum power point tracking system to minimize the energy loss in the IPMS [39]. Nonetheless, their device is PV-driven, but it was among early studies performing energy budgeting for the self-powered health monitoring system. 

Another key challenge is to effectively integrate a self-powered wearable health monitoring system with the WTEG to ensure continuous health monitoring since the power generated by WTEGs is low, and its dependency on the thermal gradient makes it even more challenging to establish continuous power to the biosensors/devices [39]. Hence, active integration is a prerequisite that can be accomplished by increasing the generated power from the TEG, reducing the power consumption of the integrated power management system (IPMS), and fabricating low-powered biosensors. The active integration between major components of a self-powered wearable health monitoring system and thermoelectric generator (TEG) modules solely depends on the energy conversion efficiency of the TEG and power consumption by biosensors, along with how conformal it is with the human skin. The conventional bulky WTEG is not compatible with soft and deformable tissues, including human skins or internal organs, which expedites the former challenges. The power requirement of biosensors is low to moderate; however, the overall system power requirement needs to be estimated and optimized. Choong Sun Kim et al. designed a self-powered electrocardiograph (ECG) monitoring system. They reported that IPMS consumed 80–90% of generated power [12]. If a highly efficient IPMS is designed, then biosensors will utilize this 80–90% generated power. Then, developing a WTEG-based health monitoring system will become even more convenient. Another evolving systematic challenge is the heating up of WTEGs during operation, most importantly for implantable TEGs [46]. Yang Yang et al. performed a study by implanting TEG modules inside a rabbit body to derive a cardiac pacemaker at low power. They successfully derived the cardiac pacemaker, though the TEG modules were heated up and aborted the operation [47]. Moreover, the design optimization of WTEGs for successful operation is still undefined and needs to be optimized. Researchers are trying to establish the governing design parameters, i.e., the number of TE legs, fill factor, film thickness, and size of device relevant to placement positions on the human body for continuous and ensured power supply from WTEGs [4,23]. Furthermore, the matching of WTEG resistances with each other, i.e., load resistance with the internal resistance and the sheet resistances of TE legs, i.e., n- and p-type, is still a challenge for WTEGs [48,49]. 

**Figure 4:**
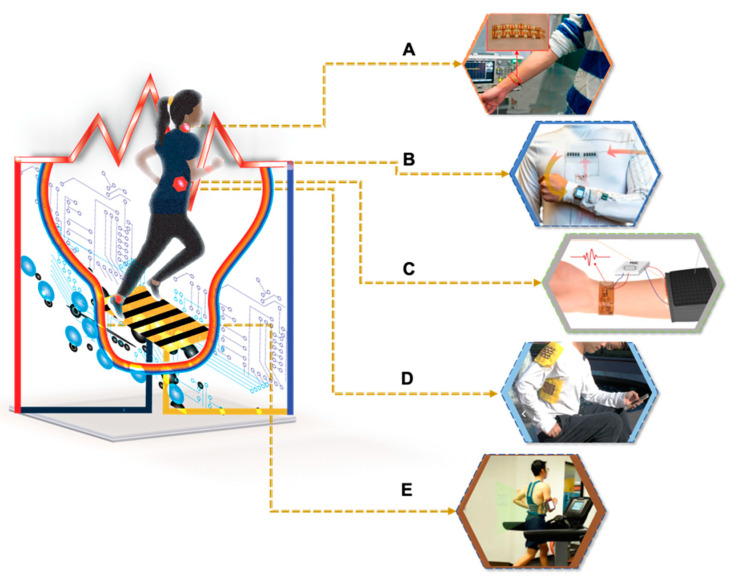
Self-powered wearable health monitoring system, powered by wearable thermoelectric generator and the prospective WTENG application on the human body: (**A**) a wearable thermoelectric generator with thermal interface material, reprinted from [45], copyright (2017), Wang, Y.; Shi, Y.; Mei, D.; Chen, Z, with permission from Elsevier; (**B**) a multi-modular microgrid energy system based on TEG, biofuel cell (BFC), solar cell (SC), and wearable applications, reprinted from [55], published under creative commons attribution-noncommercial license, nature communications 2021; (**C**) a self-powered ECG, reprinted from [12], copyright (2018), Kim, C.S.; Yang, H.M.; Lee, J.; Lee, G.S.; Choi, H.; Kim, Y.J.; Lim, S.H.; Cho, S.H.; Cho, B.J. with permission from ACSpublications; (**D**) a wearable textile-based TEG, reprinted from [56], published under creative commons attribution-noncommercial license; (**E**) a wearable application of self-powered sweat biosensor, reprinted from [57], published under creative commons attribution-noncommercial license.

In Table 1, we summarized the energy budget for frequently reported self-powered biosensors to better estimate the power requirement of self-powered biosensors. Figure 4 summarizes the material and design prospects to power the wearable health monitoring system. It collectively represents the advancement of soft electronics, biosensors, and thermoelectric materials to modify the wearable health monitoring system. However, much work is needed. For example, to the authors’ knowledge, clinical trials are not reported for WTEGs. The clinical trials of TEG-based health monitoring systems will further the real-time progress of TEGs, hence relaxing the efforts for commercializing this smart technology. Though recent studies successfully increased the efficiency of TEGs from 5% to 20% in high-temperature applications, for WTEGs, this efficiency has yet to be achieved [13,37,49,50,51,52]. The applicability of a self-powered health monitoring system needs the development of new materials, especially the n-type thermoelectric (TE) material, to increase the power output from WTEGs with the advent of flexible energy storage units and multipowered systems [53,54].

Moreover, other than systematic challenges, the cost associated with the manufacturing of commercial WTEGs is extremely high, which hampers the bulk production of WTEGs. There is a need to develop low-cost manufacturing methods for the massive production of WTEGs. The fabrication process of WTEGs involves the synthesis of powder or TE ink, fabrication of TE legs or printing of TE films, soldering of these with the interconnects, fabrication of electrodes, and, afterward, the encapsulation of the TEG device inside the polymer. Thus far, TE materials have been synthesized by using expensive synthesis techniques, such as ball milling and the hot pressing process [15,19], semisolid powder forming process [58], and sputtering process [23]. The fabrication of TE legs/films from bulk is carried out mainly through the high temperature and pressure process [15], thermoelectric material ingot processing [59], mask-assisted film fabrication [60], and 3D printing of TE films [61,62]. These processes are costly, time-consuming, and labor-intensive. Some of these even involve cleanroom processes, which further increases the manufacturing cost. Nevertheless, researchers are trying to modify the cost-effective fabrication techniques, such as the printing process to fabricate the films of TE materials, i.e., screen printing [20], blade coating [24], and 3D printing [62], for the wearable application of TEGs. Screen printing is a cost-effective process and is usually used to print the interconnects or electrodes. Thus far, screen printing of Bi-Te and CNTs has been a challenge, and very few examples have been reported in the literature. This is because the synthesis of screen-printed TE ink is still in progress. S. Liu et al. synthesized p-type SWCNT TE ink, but they only became successful in drop-casting this ink [63]. Another important challenge associated with WTEGs is the heating up of the TEG, which lowers the conversion efficiency of the WTEG. The heating up of TEG causes parasitic losses inside the TE legs/films, which destabilizes the thermoelectric gradient and negatively impacts the service life of the WTEG [21]. The stabilization of the thermoelectric gradient majorly depends on the human thermoregulatory system [64]. In the upcoming sections of this review article, the challenges related to material advances, design modifications, and system integration with biosensors/devices will be discussed.

## 4. Thermoelectric Materials

Thermoelectric converts thermal energy directly into electrical energy by employing the thermoelectric effect. A thermal gradient is developed across the TE element when a heat source is applied to one end of the TE leg, which results in Seebeck voltage across the TEG. The dimensionless figure of merit ($\mathrm{ZT}=S^{2}\sigma T/k$) measures the thermoelectric performance of TE materials [33], where S is the Seebeck coefficient, σ is the electrical conductivity, T is the average temperature, and k is the material’s thermal conductivity [20]. The higher the ZT of the TE material, the higher the conversion efficiency of TEG, and hence the higher power density [65]. The TE materials can be inorganic, referred to as all metal-based, e.g., bismuth–telluride (Bi_2_Te_3_), and organic materials, i.e., CNTs and polymers, e.g., PEDOT:PSS, polyaniline, etc. The frequently reported TE materials are listed here, i.e., inorganic thermoelectric materials, i.e., Ag-Modified Bi_0.5_Sb_1.5_Te_3_ [23], Bi_2_Te_3_ [37], Bi_2_Te_2.8_Se_0.3_ and Bi_0.5_Sb_1.5_Te_3_, Bi_2_Te_2.7_Se_0.3_, [16,31,66], and SiGe [67]; organic semiconducting material, i.e., SWCNTs [17] and MWCNTs [68]; conducting polymer-based, i.e., PEDOT nanowires [20]; and emerging TE materials, i.e., SiNW [69].

### 4.1. Inorganic TE Materials

**Figure 5:**
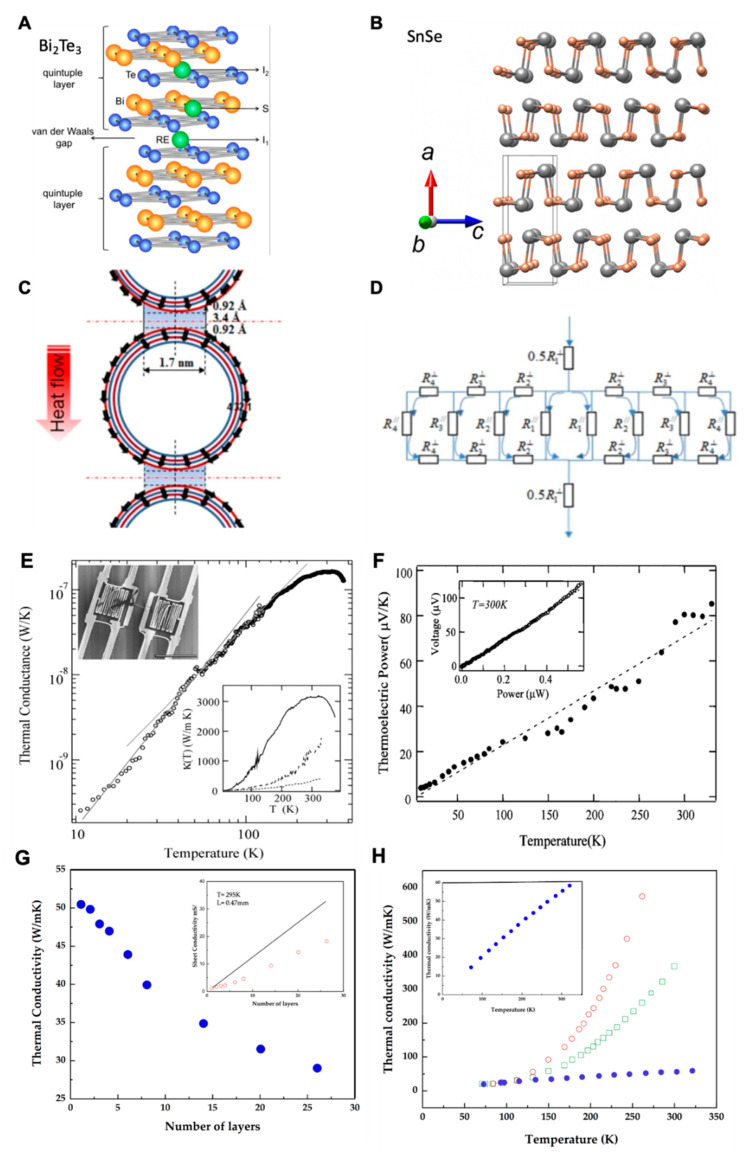
(**A**) The crystal structures of thermoelectric materials Bi_2_Te_3_ ((Bi = orange, Te = blue) reprinted from [71] published under creative common license, 2016, Scientific Reports; (**B**) SnSe (gray = Sn, yellow = Se), reprinted from [72], copyright (2018), Liu, S.; Sun, N.; Liu, M.; Sucharitakul, S.; Gao, X.P.A. with permission from AIP Publishing; (**C**,**D**) the phonon transfer and thermal conductivity phenomena in CNTs; (**C**) thermal resistances at the different layers of MWCNTs; (**D**) intra-shell and inter-shell thermal resistance network as an electrically equivalent circuit of resistors to explain the thermal resistances in CNT network, “reprinted from [80] Thermal Transport Measurements of Individual Multiwalled Nanotubes, Kim, P., Shi, L., Majumdar, A. & Mceuen, P.L., Physical Review Letters, Vol. 87, Issue 21—19 November 2001, Copyright (2019–2021) by American Physical Society”; (**E**) the thermal conductance of an individual MWNT of a 14 nm diameter. The solid lines represent linear fits of the data in a logarithmic scale at different temperature ranges. The slopes of the line fits are 2.50 and 2.01, respectively. Lower inset: solid line represents an individual MWNT (d = 14 nm). Broken and dotted lines represent small (d = 80 nm) and large bundles (d = 200 nm) of MWNTs, respectively. Upper inset: SEM image of the suspended islands with the individual MWNT. The scale bar represents 10 mm; (**F**) thermoelectric performance of CNTs at different ΔT: inset is the plot between output power and output voltage, reprinted from [80], copyright(2001), Kim, P.; Shi, L.; Majumdar, A.; McEuen, P.L., with permission from Physics Review letters; (**G**) variation in thermal conductivity of MWCNTs based on the number of layers; (**H**) variation in thermal conductivity of MWCNTs based on the length 7.6 mm (open circles), 5.4 mm (open squares), and 0.37 mm (solid circles). Inset shows the expanded plot for L = 0.37 mm sample, reprinted from [83], copyright (2007), Aliev, A.; Guthy, C.; Zhang, M.; Fang, S.; Zakhidov, A.; Fischer, J.E.; Baughman, R.H., with permission from Elsevier.

Bismuth antimony telluride (Bi-Sb-Te) is a 2D inorganic material. It builds on stacked atomic layers, densified internal microstructures, and shortened grain boundaries. It is considered a suitable candidate for TEG because of its good thermoelectric performance; however, flexibility is a challenge for this material [23]. In recent years, its ZT has been reported as ~0.8 at T_h_ = 250 °C − 1.4 at T_h_ = 25 °C at ΔT = ~3 K–225 K), the highest among all TE materials [19]. This high value of ZT is due to low thermal conductivity and high electrical conductivity that assist in the transfer of electrons during the thermoelectric process [70]. Figure 5A,B show the atomic structure of 2D bismuth–telluride (Bi_2_Te_3_) and tin selenide (SnSe) [71,72]. The higher electrical conductivity of these materials is due to the higher charge carrier concentration, higher mobilities, and strong long-range interaction in bismuth (Bi) and antimony (Sb) along a specific crystallographic direction. These specific properties collectively control the transport of electrons and phonon inside the material. Lee et al. applied first-principles calculations to study the phonon transport in Bi and bismuth antimony (Bi-Sb) alloys. They found that long-range bonding originated the resonant bonding, which resulted in lower thermal conductivity and softening of the optical phonons. It helped scatter the acoustic phonons responsible for thermal conductivity. Conclusively, the diffusive flow of phonons in Bi-Sb-Te yielded a high ZT. These Bi-Sb-Te films work as a p-type material [73]. Previously, disruption in the electrical conductivity of bismuth telluride-based alloys caused an irregularity in energy generation. Considerable efforts were done to modify the atomic structure of bismuth telluride-based systems to regularize the TE performance, such as introducing defects. These defects enhanced the electrical conductivity of Bi-Te material and increased the ZT [33]. Deyue Kong et al. fabricated a magnetron-sputtered (Bi_2_Te_3_) n-type film-based flexible WTEG and studied its in-plane performance at room temperature. They found that the Bi_2_Te_3_ film grown in the (00l) plane at 3 Pa pressure exhibited the highest Seebeck coefficient and a high-power factor at room temperature (RT) (Table 2). The controlled microstructure and in-plane crystallinity added more crystalline boundaries to phonon scattering. As a result, the Seebeck coefficient increased from ~ −148 to ~ −177.2 µV/cm·K^−2^, as did the power factor from 11.75 µW/cm.K^−2^ to 21.6 µW/cm.K^−2^ [15]. Hongjin Shang et al. developed a p-type flexible Ag-modified Bi_0.5_Sb_1.5_Te_3_ thermoelectric generator. The silver (Ag)-doped Bi_0.5_Sb_1.5_Te_3_ films improved electrical conductivity from 1.2 × 10^4^ S/m to 7.5 × 10^4^ S/m, the Seebeck coefficient from 89.20 µV/K to 128.98 µV/K, and the power factor from 0.977 µW/cm·K^−2^ to 12.4 µW/cm. K^−2^, at RT (Table 2). The Ag doping triggered this increment in the performance by enhancing the carrier concentrations and improving the carrier mobilities [23]. Their study revealed that adding structural defects in the material would add to the carrier transport, leading to a higher figure of merit ZT. 

In another study, Hwanjoo Park et al. designed a watch strap-based WTEG using a bulky TE material that generated output power of 93.72 mW at a ΔT of 1.43 K in a walking state at an exceptionally low thermal contact resistance of 0.005 m^2^K/W [16]. However, it is bulky, inflexible, and difficult to fabricate. Furthermore, it inherits the rigidity and brittleness of metals, impacting its integration with smart electronics. Bed Poudel et al. synthesized a nanostructured p-type Bi_2_Te_3_ alloy using ball milling and hot-pressing processes. They reported a ZT of 1.02 at room temperature. They stated that the nanograins’ formation of nanodots and uniformity in the Bi_2_Te_3_ material during the ball milling process helped increase the ZT value [19]. Although Bi-Sb-Te-based TE systems exhibited excellent thermoelectric properties and high-power factors, these films lack flexibility, which reduces their scope for WTEGs. Therefore, researchers successfully overcame the flexibility challenge of these materials. They fabricated the films of the Bi–Sb–Te system and embedded them into polydimethylsiloxane (PDMS). Because of the mismatch of Young’s modulus of Bi-Sb-Te with PDMS, however, the inherited rigidness did not provide good conformality on curved human curvatures, i.e., the neck, around the side of the diaphragm, and upper arm. Thus far, researchers have only been successful in presenting a wrist-wear WTEG at lab scale. Another challenge associated with the low thermal conductivity of the Bi–Sb–Te system is the formation of heat spots inside the TE legs, which causes a lag in the performance of the WTEG. The Bi–Sb–Te system is considered the best TE system. However, because of the low thermal conductivity, these TEGs need an engineered heat sink on the cold side to ensure their proper functioning [12,21]. For commercial applications, this is an important challenge to address. A WTEG device needs to be flexible, stretchable, and conformable to human skin to absorb and convert maximum body heat into electrical energy. Yet, the Bi–Sb–Te system is rigid and cannot offer the required scalability, flexibility, and conformity to human skin, making it ineligible at present. The difficulty of miniaturization lowers their demands and urges the need for innovative materials that are more flexible yet exhibit higher TE conversion efficiencies at ΔT = ~3 K [16,65,66].

### 4.2. Carbon-Based TE Materials

Carbon-based TE materials are lightweight, flexible, and durable. Recent studies reported good thermoelectric properties of carbon nanotube (CNT)-based TE films. Especially after the advent of 1D carbon nanotubes (CNTs) by Iijima in 1991, CNTs have become the materials of energy transfer and storage [77]. The latest research is focused on implementing the magic of this versatile material in the energy field. CNTs exhibit different band gaps and represent a unique one-dimensional energy flow that satisfies the Thomson effect to enhance the TE efficiency (Figure 5C,D) [41]. CNTs are multi-walled or single-walled [30,62]. Single-walled carbon nanotubes (SWCNTs) can offer good thermoelectric properties because of their excellent electrical conductivity. However, the thermal conductivity of individual SWCNTs (10, 10) is high, i.e., 6600 W/mK, limiting their TE performance in the individual form [78]. For an individual multi-walled carbon nanotube (MWCNT), the thermal conductivity is ~3000 W/mK, and S = 80 µV/K at a room temperature [79,80]. However, recent studies reported very low thermal conductivity values for a bundle/pellet of SWCNTs and films/bed of SWCNTs, i.e., ~5 W/mK [81] and ~0.15 W/mK, respectively [82]. These low values of thermal conductivity are due to the high thermal contact resistances in the composite at the inter and intra-shell level (Figure 5C,D), which are higher in the smaller tubes of diameter, i.e., <1–2 nm, which limits the energy transfer in Van der Waals contact because of the packed junction and weak linkage of the SWCNTs. However, this results in a reverse effect on the electrical conductivity, which is ~3–5 × 10^3^ S/cm for a bundle of SWCNTs. At these values, the corresponding ZT was calculated at 0.2 without doping or altering the properties of SWCNTs [82]. The presence of impurities, contact pads, disorder arrangement of SWCNTs, and structural defects lower the phonon transmission in the bulk material, hence further lowering the thermal conductivity [81].

Kim et al. employed a simplified thermal conductivity model to study the effect of temperature on the thermoelectric performance of the MWCNTs. Figure 5E shows that an increase in temperature increases the thermal conductivity of the MWCNTs to 120 K. However, the curve declines after this. Figure 5F shows a linear increase in the thermoelectric power curve to 300 K, which is a good feature of the MWCNTs. The decrease in thermal conductivity helped establish the thermal gradient across the length of MWCNTs, resulting in a linear rise in thermoelectric power. They found that MWCNTs followed the power law with an exponent of 2.5 at low temperatures from 8 K < T < 50 K [80]. 

In this range, low-energy phonons dominate the thermal conductance. As the temperature rises, the concentration of high-energy phonons increases with an increase in carrier mobilities. However, with a further rise in temperature after 320 K, the phonon mean-free path decreases, causing a decrease in thermal conductivity. However, it increases the electrical conductivity of the MWCNT, which raises the power factor [80]. Aliev et al. performed extensive research to understand the variation in the thermal conductivity of MWCNTs based on the structure and order of MWCNTs. They reported that the length of MWCNTs is not a significant factor for a temperature ≤ 150 K. They found that the thermal conductivity for the MWCNT sheet is lower than the individual MWCNT (Figure 5G,H). In MWCNT sheets/bundles, the structural defects and layer-to-layer heat transfer are ineffective compared to freestanding MWCNTs. Moreover, in individual MWCNTs, optical phonons are responsible for the heat conduction, which becomes scattered in the MWCNT sheet [83]. The thermal transport in horizontally aligned MWCNTs is higher in the in-plane direction. However, the out-of-plane thermal conductivity and thermal contact resistance in horizontally aligned MWCNTs are lower [30]. However, lower electrical conductivity emphasizes modifying the thermoelectric characteristics of MWCNTs. Fortunately, sp2 hybridized CNTs have defects and impurities on the surface, i.e., carbon residue, oxygen impurities, residual catalysts, etc., which can be of assistance in increasing the TE performance [63,84]. SWCNTs are sp2 planar hybridized and offer a direct flow to electrons/holes to transfer from one end to the other end. This unidirectional flow enhances the electrical conductivity of the SWCNTs and adds to the thermal conductivity. However, in SWCNT pellets or films, the thermal conductivity decreases, which enhances the thermal power to many folds. The latest research on SWCNTs demonstrated that the thermal conductivity of SWCNTs could be reduced further by fabricating an organic–polymer composite. There are many conducting polymers that exhibit very low thermal conductivity, i.e., poly (3,4-ethylenedioxythiophene) polystyrene sulfonate (PEDOT:PSS) [63], polyaniline (PANI) [85], polyethyleneimine (PEI) [86], polyurethane (PU) [62], etc. The high thermal conductivity of SWCNTs is advantageous to the thermoelectric at room temperature since it does not assist in the formation of heat islands inside the TEG; moreover, it does not require a heat sink to remove excessive heat from the WTEG. The most important attribute of SWCNTs is that the SWCNT-based TEGs are air-stable and do not need sophisticated encapsulation to function [17]. Yoshiyuki Nonoguchi et al. synthesized air-stable n-type SWCNT film and reported a high *S* of −63 µV/K at T = 3 × 10 K [74].

### 4.3. Organic-Inorganic and Hybrid Matrix

**Figure 6:**
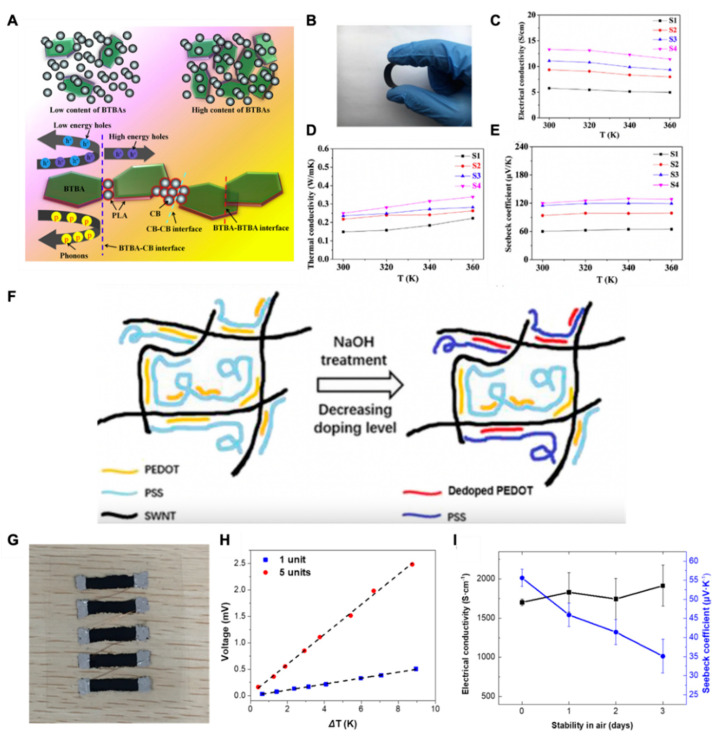
(**A**) The improvement in the hole transfer in Bi_2_Te_3_ by the addition of CB particles; (**B**) the camera image of hybrid Bi2Te3/CB film; (**C**) corresponding electrical conductivity at various temperatures; (**D**) corresponding thermal conductivity at various temperatures; (**E**) corresponding Seebeck coefficient at various temperatures, reproduced with permission from [61], published under creative common license for open access; (**F**) schematic illustration of de-doping process of PEDOT:PSS by NaOH treatment to enhance the electrical conductivity; (**G**) camera images of the 0.1 M NaOH-treated SWNT-60 thermoelectric modules on PET substrate; (**H**) the voltage generated by the TE modules as a function of ΔT; (**I**) TE air stability of 0.1 M NaOH-treated SWNT-60 nanocomposites, reprinted from [63], copyright (2019), Liu, S.; Li, H.; He, C. with permission from Elsevier.

Recently, it has been reported that the thermoelectric performance of TEG increased by using assisting materials, i.e., the conducting polymer and highly electrically conductive carbon black CB, and by introducing Bi_2_Te_3_ with CNTs, and vice versa [22,62,68,75,87]. Young Du et al. studied the effect of carbon black on the thermoelectric performance of a Bi_2_Te_3_-based thermoelectric generator. They prepared a composite of CB/bismuth telluride-based alloy/additive (BTBA). They found that ~10–20 wt% of carbon black increased the ZT value of the composite from 0.004 to 0.023 at room temperature. The higher electrical conductivity of CB and the low thermal conductivity of the conducting polymer collectively improved thermoelectric efficiencies at room temperature, as shown in Figure 6A–E [61]. Tzounis L et al. reported an excellent performance of a 3D printed MWCNTs and polyurethane (PUE) composite. They stated that the anisotropic 3D printed PUE/L-MWCNTs (5 wt%) generated the maximum power factors of 0.02 μW/mK^2^ and 0.04 μW/mK^2^ through-layer and cross-layer directions, respectively. It was noted that the cross-layer thermoelectric performance was better for MWCNTs than the through-layer. The multilayers of C_60_ scattered the phonons at a higher rate, providing an edge for electrons to transfer at shorter distances [62]. Marcin Sloma et al. used mechanical mixing and in situ polymerization to fabricate a nanocomposite of polyaniline (PANI) and MWCNTs. They found that mechanical mixing of MWCNTs with the conducting polymer PANI improved the TE performance. The nanocomposite film exhibited the thermoelectric parameters, i.e., electrical conductivity, Seebeck coefficient, and power factor of 405.45 S·m^−1^, 15.4 μV·K^−1^, and 85.2 nW·m^−1^K^−2^, respectively. The dielectric layer of PMMA provided a bridge for the electrons to flow, which increased the power factor for the nanocomposite [75]. Figure 6F–I show the enhancement of electrical conductivity and the Seebeck coefficient for the SWCNTS/PEDOT:PSS composite [63].

## 5. Thermoelectric Challenges in CNTs

**Figure 7:**
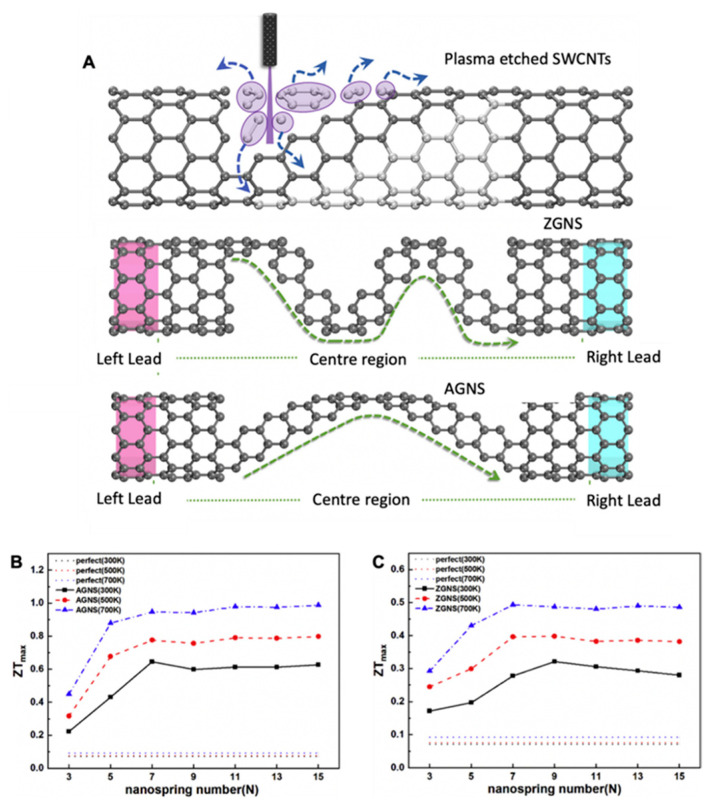
(**A**) Schematics of plasma-etched SWCNTs; thermoelectric ZGNS-modified SWNTs; AGNS-modified SWCNTs; (**B**,**C**) ZT of AGNS-modified SWCNTs in comparison with pristine CNTs, reprinted from [88], copyright (2020), Xu, X.; Xiao, H.; Ouyang, T.; Zhong, J. with permission from Elsevier.

Nguyen T. Hung et al. reviewed the effect of geometric parameters, i.e., the concentration of doping, diameter, electronic structure, length, and the thickness of SWCNT sheet, and operative parameters, i.e., the temperature on the thermoelectric performance of SWCNTs. In their study, although the thermal conductivity of SWCNTs was high after modifying the doping level of semiconducting SWCNTs, the power factor increased from 700 to 1000 µW/mK^2^, roughly equal to the PF of Bi_2_Te_3_, though the ZT value for SWCNTs is 0.05 [41]. However, to obtain a high value of ZT, the thermal conductive properties of SWCNTs need to be altered and studied [41], which is extremely low compared to the 1.2 ZT value of Bi_2_Te_3_ at 300 K [19]. The comprehensive review of the thermoelectric performance of SWCNTs shows that regulating the doping mechanism brings a significant increase in power factor. Xiaoyan Xu et al. incorporated graphene springs inside the CNT film to increase the ZT value for CNTs. They studied armchair and zigzag patterned nano springs and optimized the number of nano springs for good ZT. The modified CNT film with zigzag graphene nano springs (ZGNS) increased the ZT from 0.05 to 0.3 for seven ZGNS at room temperature. Furthermore, for armchair graphene nano springs, they even found that ZT = ~0.64, which is a remarkable improvement in the thermoelectric properties of CNTs. Figure 7 shows their modified CNT network [88]. Henceforth, it is advisable to synthesize hybrid thermoelectric devices either by doping or by introducing another TE material. The inherited high electrical conductivity, flexibility, and mechanical strength of SWCNTs and low thermal conductivity of inorganics and polymers will collectively expedite the thermoelectric efficiency. Nevertheless, with material advancement, TE efficiency can be increased. In particular, the increase in the Seebeck coefficient decreases the electrical conductivity, which requires further research [48,50].

## 6. TE Systems Architecture and Design

The design of the TE system consists of interconnects to bridge the p-type leg with the n-type leg electrically and electrodes to connect with the power management unit. The thermoelectric performance of the WTEG is highly dependent on the number of TE pairs since it determines the number of interconnects. Undoubtedly, an increase in the number of pairs will add up the output power, but an increase in the number of interconnects will also add up the internal resistance, and hence will hurt the overall TE conversion efficiency [89]. Most TE systems employ metallic interconnects irrespective of the TE material, whether it is a carbon-based or metal-based TE system. Although the metallic interconnects assist the metal-based TE systems, they will have a reverse effect when they are employed with carbon-based material [17]. The interconnects of silver (Ag) [21]; interconnects of copper (Cu) [25]; liquid metal-based, i.e., eutectic gallium–indium (EGaln), interconnects [90]; and liquid metal-embedded elastomer (LMEE) interconnects [91,92] are frequently reported in the literature. In recent years, LMEE interconnects have been more attractive since they offer better flexibility than metallic interconnects. Moreover, their stretching and under strain performance does not vary much compared to metallic interconnects [92]. However, TE legs must be optimized to increase the TE efficiency. Table 3 compares the thermoelectric performance of various TE system architectures. In a subsection, we will discuss the effect of geometric parameters and the conformality of the TEG.

### 6.1. Effects of the Geometric Parameters on TE Performance

The performance of WTEGs is not only dependent on TE material properties, but it also depends on the geometric parameters of the WTEG. Unfortunately, thus far, design optimization has not been done and needs exploration. It is noteworthy that an important design parameter, i.e., fill factor, should be studied so that WTEG research can progress. For instance, Shifa Fan et al. studied the effect of fill factor, leg shape, leg height, and the number of TE legs on the thermoelectric performance of the Bi_2_Te_3_-based TEG. Their experimental and numerical study showed that the output power was higher for tri-prism-shaped TE legs than for cuboid, cylindrical, and hexagonal prism-shaped. However, the mechanical stress was 222.7% higher than the mechanical stress for the cuboid leg shape. Moreover, the output power of the cuboid shape was only 1.22% less than the tri-prism-shaped TE legs. Furthermore, they reported that the maximum output power reached 90.98 mW at a fill factor of 0.64, a leg height of 0.5 mm, and the number of TE legs = 64. Hence, a WTEG should be designed accordingly [96]. Ji Dongxu et al. found geometric parameters similar to those of Shifa Fan et al. and agreed with the findings of their study [97].

### 6.2. Conformal Contact with Human Skin

WTEG harvests body heat and converts it directly to a useful form of electrical energy at a reasonable thermal gradient. The successful stabilization of a thermal gradient requires the WTEG to be in conformal contact with human skin. Presently, WTEGs are mostly based on inorganic materials, i.e., Bi_2_Te_3_ [15]. These inorganic materials are best in the TE field; however, these are unable to provide conformal contact with human skin. This is due to the mismatch of the strain tolerance between human skin and inorganic material. Human skin is soft in nature with high mechanical strength; however, inorganic materials are rigid in nature [21]. A recent attempt has been made to resolve this predominant challenge of WTEGs by introducing polymer-based TE materials and encapsulations. Researchers are improving the conformal characteristics of the Bi_2_Te_3_-based WTEG by encapsulating the TE module in flexible and stretchable substrates, such as PDMS and Ecoflex. For example, Wei Ren et al. improved the stretchability of Bi-Sb chalcogenides by using liquid metal wiring to 120% in indoor and outdoor environments [31]. However, the bulkiness of the WTEG still needs to be addressed. Byeongmoon Lee et al. introduced soft magnetic silver-coated Ni particle heat conductors in the PDMS matrix to improve the conformity of the Bi_2_Te_3_-based WTEGs and eventually enhanced the flexibility [21]. Although they achieved a 7.02 mW output power at the ΔT = 40 K for the 120 µm thick encapsulating layer of PDMS, the bulkiness of the TEG is still a challenge. Carbon-based TEGs offered good conformal contact because of inherited mechanical strength and flexibility, i.e., MWCNTs and SWCNTs. Kyung Tae Park et al. reported 1.65 µW at a ΔT = 30 K from 60 n–p pairs of SWCNT thermoelectric bracelets [29]. Qian Wu et al. fabricated a 3D fabric based WTEG of MWCNTs and reported that the prepared fabric conforms well with the human body. It generated a voltage of ∼800 μV and output power of ∼2.6 nW at ΔT = 66 K [98]. Therefore, the carbon-based TEGs do not have higher power densities than Bi–Sb–Te TEGs. However, these WTEGs offer the best conformal contact with human skin, hence increasing the strain tolerance of WTEG.

## 7. Future Perspective for WTEG Design and Performance

**Figure 8:**
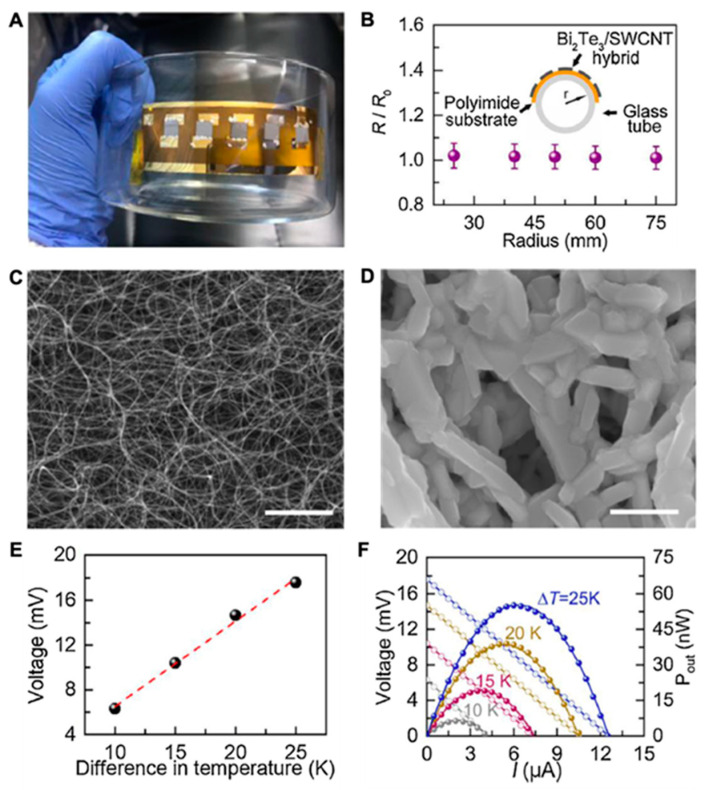
A flexible thermoelectric device based on hybrid Bi_2_Te_3_–SWCNTs. (**A**) The camera image of the WTEG device; (**B**) the bending performance of WTEG at different tube radii; (**C**) SEM image of SWCNTs; (**D**) SEM image of Bi_2_Te_3_–SWCNTs hybrid; (**E**) the output voltage of the hybrid Bi_2_Te_3_–SWCNTs at different ΔT; (**F**) the corresponding thermoelectric performance of the hybrid Bi_2_Te_3_–SWCNT-based WTEG, reprinted from [22], copyright (2020), Li, Y.; Qiao, J.; Zhao, Y.; Lan, Q.; Mao, P.; Qiu, J.; Tai, K.; Liu, C.; Cheng, H. with permission from Elsevier.

**Figure 9:**
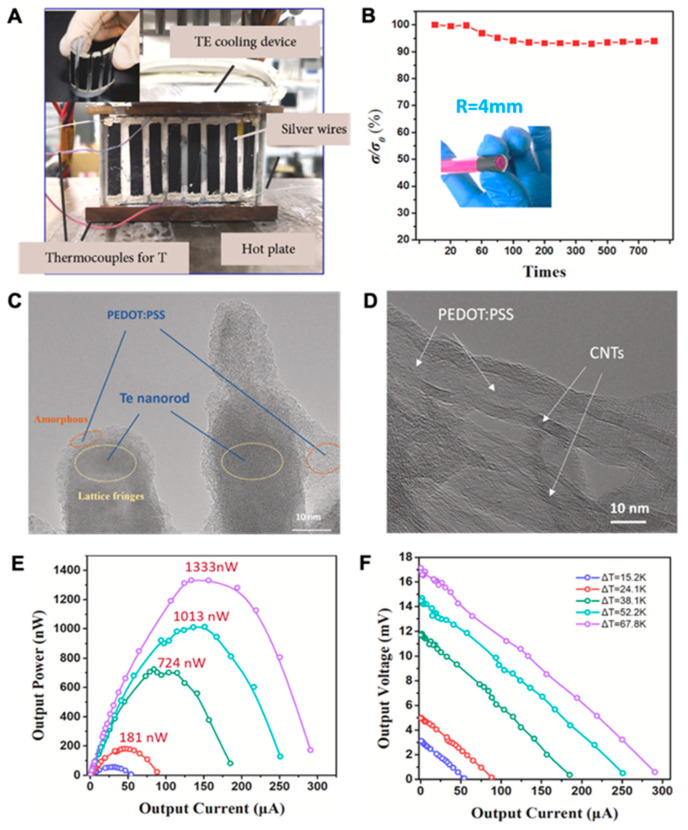
(**A**) the camera image of the thermoelectric device during testing; (**B**) the bending test of the device with 60% of CNTs; (**C**) the SEM image of PEDOT:PSS–Te; (**D**) the SEM image of PEDOT:PSS–CNTs; (**E**) the thermoelectric performance of the hybrid WTEG; (**F**) the open-circuit voltage and short circuit current at different ΔT, reprinted from [99], copyright (2021), Liu, C.; Shan, D.-L.; Shen, Z.-H.; Ren, G.-K.; Wang, Y.; Zhou, Z.-F.; Li, J.-Y.; Yi, D.; Lan, J.-L.; Chen, L.-Q.; et al. with permission from Elsevier.

The conformity and flexibility of the WTEG are desired attributes for building a good interface with human skin. Given the bulkiness of Bi_2_Te_3_-based WTEGs and the low power density of carbon-based WTEGs, the present research has been focused on developing hybrid WTEGs. Yin et al. prepared a 180 nm thin-film hybrid WTEG of Bi_2_Te_3—_SWCNTs and reported a remarkable performance with only 1.4 KΩ of internal resistance. The output voltage and power were 17 mV and 55 nW at ΔT = 25 K, respectively [22]. Their device showed excellent mechanical performance with only a 4% change in R_0_ during the bending test and good electrical stability after 200 bending cycles (Figure 8). Liu et al. studied the role of interfaces in hybrid TENG. They found that at the interface of polymer–inorganic, an extremely low thermal conductivity of 0.39 W/mK at 410 K resulted in excellent output power of 1.33 μW [99]. In their study, the high interface thermal resistance and interface density at PEDOT:PSS–Te and PEDOT:PSS–SWCNTs collectively became the barrier for phonon transport, actuated the phonon scattering, and improved the performance of the hybrid WTEG (Figure 9). However, the hybrid WTEG was bulky in size and needed further modification for the flexibility and miniaturization of the WTEG.

The conformal contact of WTEG with human skin requires building a good interface, which helps to build up the thermal gradient. The interface between WTEG and human skin is composed of different layers: (1) human upper skin, which has different thermal resistance and Young’s modulus, and (2) bottom layer of the WTEG, e.g., PDMS/Ecoflex. Both layers have a different strain tolerance and Young’s modulus to offer a good interface. It is very challenging to bring the mechanical properties of these two layers close to each other without impacting the performance of the WTEG. The bulky TEGs need a thick layer of packaging, which destabilizes the match between the Young’s modulus of human skin and TEG without lowering the output power. Soft packaging and film-based designs are advantageous for overcoming these challenges. Future research should be focused on addressing the mismatch of the Young’s modulus of human skin with the packaging layer and TE materials. An important technology to address interface challenges is the epidermal electronics system (EES) [100]. According to this, conformal contact depends on the interface mechanics, adhesion to soft tissues on the human skin, strong Van der Waals forces at the interface, and the Young’s modulus of human skin and the wearable device [101]. These interfacial challenges have not been explored yet for WTEGs. EES will help to improve the adhesion of WTEGs to the human skin. The assessment of device performance has not yet been done. There have been many examples reported of the successful working of a WTEG on the human body, but clinical trials to assess the device’s performance in a real-time environment have not yet been done [12,21]. This is due to the above-mentioned challenges that have hampered the clinical trials of WTEGs. Future research on WTEGs should include clinical trials for better assessing the TE materials, design, interfacial contact, conformity, flexibility, and life cycle of self-powered biosensing devices.

## 8. Self-Powered Health Monitoring System

**Figure 10:**
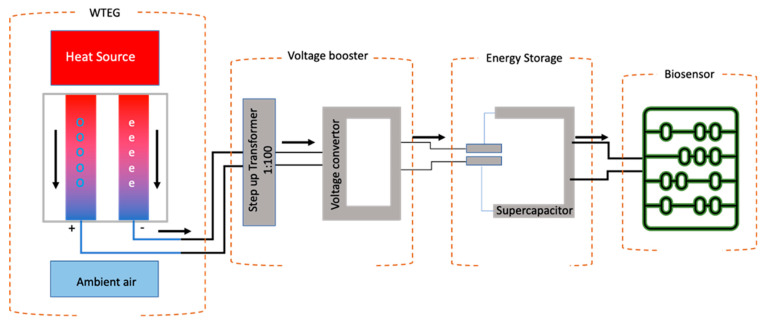
The schematic representation of a self-powered health monitoring system, referenced from [12].

A self-powered health monitoring system is comprised of the following electronics components: a direct energy supply, e.g., WTEG; voltage booster; voltage regulator; energy storage device; and biosensor-based health monitoring system (Figure 10). These components need to be compatible for the efficient function of self-powered health monitoring. In reviewing the prospects of thermoelectric generators for harvesting human energy, Amin Nozariasbmarz et al. highlighted the importance of design and recommended modifying the architecture of integrated power management systems (IPMS). Since the current available IPMS were designed to power Li-ion batteries, they correspondingly exhibit less compatibility with WTEGs. Furthermore, for a WTEG, the efficiency of a voltage booster is a deciding factor because a low voltage startup is necessary to start the IPMS [2,12]. In addition, the fluctuations in the output voltage and charging/discharging cycles of WTEGs are different from batteries because the WTEG is entirely dependent on the operating environment and the activities of the user [2,102,103,104]. Hence, integrating the WTEG with health monitoring systems is a prerequisite to modifying the design of IPMS. The design and material of biosensors need to be studied further. The most crucial factor to be powered by a WTEG is the power consumption of the health monitoring system. The current architecture and materials of biosensors make the integration of the WTEG with modern/smart electronics more difficult. Therefore, the power consumption of the health monitoring system needs to be lowered. An energy budget audit of the wearable self-powered health monitoring system will help to address the following challenges.

## 9. Integrating the WTEG with Biosensing Devices

The current challenge of WTEGs is the conformal contact of the WTEG with human skin, which is yet unachievable. If the WTEG design is modified by employing soft electronics to build a flexible power management circuit, then a corresponding increase in power density will be observed. Recent advances in flexible electronics will help to revolutionize the WTEG. Researchers successfully developed soft electronics-based biosensors to enhance signal optimization and improved the diagnostic efficiency up to many fold [46,53,54,105]. The bio-polymer-based soft electronics offered biocompatibility and eligibility to increase energy generation. Hojoong Kim et al. developed a soft bioelectronic system comprised of a multilayered flexible circuit mounted on the top of the biosensor to increase the signal optimization [106]. This soft sensor technology can resolve the challenge of system integration with the biosensors at high efficiency. However, these aspects need to be explored and experimented with. In addition to flexibility, energy dissipation is another challenge. In recent years, capacitor/supercapacitors have received more attention. They have successfully been applied in the IPMS of self-powered systems to reduce energy dissipation effectively. Jinfeng Yuan built a fully self-powered wearable monitoring system using capacitors and achieved low energy consumption with high sensitivity [4]. The capacitor/supercapacitor can resolve the challenge of an irregular energy supply from the WTEG. It will store energy during idle mode and provide power during the working cycle of the IPMS. This will shorten the start time of the sensors and the overall energy system. Moreover, supercapacitors consume less energy compared to lithium-ion batteries. Another option is to consider capacitors for boosting up and regulating the voltage from the WTEG; then, this voltage can be stored in a coin cell battery or in a supercapacitor to derive the biosensor unit [93]. Brian Iezzi et al. developed a supercapacitor-based industrial monitoring system and successfully performed wireless temperature monitoring [107].

For addressing the system integration and incorporating the whole wearable with flexibility, their study is worth applying in the field of WTEGs. Notably, a detailed energy consumption analysis of each component is recommended to better estimate the efficiency of WTEGs. For example, it is expected that in an idle state, the biosensor system will not consume power. Conversely, the microcontroller unit (MCU) and Bluetooth system consumed a surplus amount of power in standby mode, as presented in Table 4. For instance, TICC2650 MCU consumed 10 mW of power in an active state during gesture recognition [39,108]. If this MCU is replaced by an ultra-low power MCU, then a fraction of power can be saved to operate the biosensor unit without interruptions. For example, STM32L476RG consumes only 1 µW in the standby position, and it consumes only 171 µW (LDO mode) in a running state, which is far less than the TICC2650 MCU [4,109]. Another important task is to lower the power consumption of Bluetooth low energy (BLE) devices to perform the signal transmission at the receiver. The power requirement of biosensors varies from 5.4 µW for a micro-accelerometer to 2 mW for wireless biosensor systems, i.e., an accelerometer + temperature sensor + humidity sensor [4]. All these biosensors have been powered by human body heat [4,12,39]. If an IPMS is equipped with a microwatt BLE, then this power requirement will be sufficiently reduced. As mentioned, the BLE system utilized 4 mW during the signal transmission of gesture recognition to the receiver, which is a drastic amount of power to be used [39]. Based on statistics, low-powered BLE devices need to be explored and employed [39]. If nano/micro-powered low-power BLE systems are incorporated in the IPMS, then more energy can be saved, i.e., PSoC™ 4 BLE. This BLE-equipped microcontroller unit (MCU) has a power requirement of a few microwatts [110]. Thus, affordable self-powered health monitoring systems will become a reality.

**Figure 11:**
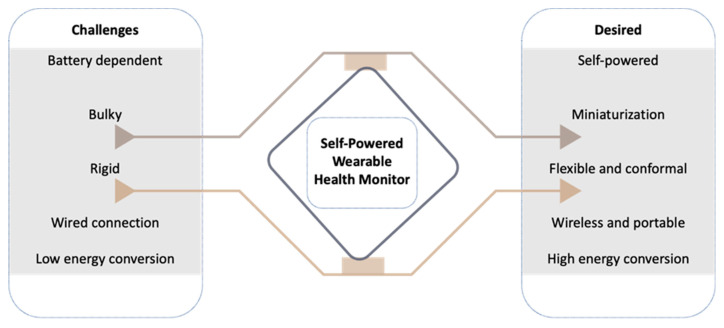
A brief overview of challenges and future perspectives for self-powered health monitoring systems.

Hence, to resolve the challenges of thermoelectric generators, the exploration of new TE materials, an innovative design strategy, and a low-power IPMS are needed. The design aspects of WTEGs should consider the TE material, a number of n and p pairs, device’s orientation, flexibility, and conformal contact with the human skin to harvest good TE power (Figure 11). The current literature database regarding the integration of WTEGs with biodevices/wearable electronics has yet to be explored because very few pieces of literature are available for this proceeding. To enhance the biomedical application of thermoelectrics, clinical trials should be done so that the real-time accuracy of the WTEG performance can be optimized. The scope of WTEGs can be increased by reducing the manufacturing cost. At present, the lab-scale fabrication of WTEGs is successfully performed using costly fabrication techniques. However, the bulk production of WTEGs needs cost-effective and fast fabrication processes to commercialize the technology. Considerably, wet chemical material synthesis and fast printing processes would be fruitful in fabricating low-cost WTEGs.

## 10. Conclusions

This review highlights recent advancements and challenges with materials and WTEG-enabled self-powered health monitoring systems. A WTEG has been introduced to power various wearable sensors and electronics, which minimizes their dependency on conventional energy sources, such as batteries. An integrated wearable electronic system with a WTEG offers continuous, real-time monitoring of the human body’s physiological signals while miniaturizing the device’s form factor without using batteries. Thus, this system considerably enhances the accuracy of wearable diagnostic sensors, as opposed to conventional health monitors that only measure data on several occasions. However, additional technological advancements are necessary to improve the thermoelectric performance and reliability of a WTEG. The combined studies of nanomaterials, printing techniques, strain-isolating mechanics, and soft packaging methods will help develop more accurate, low-profile WTEG biosensors and bioelectronics.

## Figures and Tables

**Table 1:** Energy budget for WTEG-based self-powered biosensors.

Energy Source	Energy Harvester	Power Density	Sensor	Power Required for Biosensor	Reference
**Human Body Heat**	w-TEG	38 (µW/cm^2^)	ECG	<15 (µW)	[12]
**Solar**	PV module	2.8 (mW/ cm^2^)	Accelerometer + gyroscope	1.3–4.3 (mW)	[39]
**Human Body Heat**	f-TEG	3.5 (µW/cm^2^)	Accelerometer + temperature + humidity sensor	2 (mW)	[4]
**Human Body Heat**	w-TEG	0.5 (µW) (no area information is given in this reference)	Temperature and strain sensor	(10 µW)	[53]

**Table 2:** Room temperature thermoelectric properties of inorganic and organic thermoelectric materials for film-based WTEG.

Materials	σ (S/m)	K (W/m·K)	S (µV/K)	Film Thickness (nm)	Power Factor (µW/cm·K^2^)	ZT	Reference
Bi_2_Te_3_ (n-type film)	6.9 × 10^4^	-	−177	800	21.7	0.05	[15]
Bi_0.5_Sb_1.5_Te_3_ and Bi_2_Te_2.7_Se_0.3_	5.95 × 10^4^	1.03	211.15	-	-	-	[16]
Ag-Modified Bi_0.5_Sb_1.5_Te_3_	7.5 × 10^4^	-	128.98	750	12.4	-	[23]
Bi-Te alloy (nanodots)	1.35 × 10^5^	1.157	186.54	-	~43	1.2	[19]
L-MWCNTs/PU (12–25 µm)	133.1	12–17	19.8	-	0.0004		[62]
n-type SWCNT film (in-plane TE)	2.05 × 10^3^	39 ± 12	−33	-	2.3	0.002	[74]
n-type SWCNT film (through-plane TE)	69	0.12 ± 0.001	−63	-	0.27	0.07	[74]
PANI/MWCNTS	405.45	-	15.4	-	852 × 10^−6^	-	[75]
SWCNTs/ Bi_2_Te_3_	1.1 × 10^4^	0.33	−152.1094	180	2.03	0.23	[22]
As grown SWCNT film	3.02 × 10^5^	-	78	200	18.40	-	[18]
SWCNTs/PEI (1 wt%)	3.63 × 10^5^	-	−64	200	15	-	[18]
SWCNTs/PANI	4 × 10^5^	-	17	-	1.0	-	[76]
SWCNTs/PEDOT:PSS	17.01 × 10^4^	0.4–0.6	55.6		5.26	0.39	[63]

**Table 3:** Analysis of WTEGs based on organic and inorganic thermoelectric materials.

TE Architecture	TE Materials	ΔT(K)	Open Circuit Voltage	Power Density	Main Features	Application	Reference
Conformal film Lego-thermo-electric chips 112 TE legs	Bi_0.5_Sb_1.5_Te_3_ and Bi_2_Te_2.8_Se_0.3_	95 and 93	1 V/cm^2^	19 μW/cm^2^	Self-powered, Self-healing, Stretchable-120%, Recyclable, Eco-friendly	Various targets	[31]
Conformal, magnetic soft-connect 440 TE legs	Bi_2_Te_3_, silver-coated nickel (Ag–Ni), Ag nanowires	3–6	266 mV	6.96 μW/cm^2^	Self-powered, Flexible, Stretchable-20%	Wearables	[21]
Thermoelectric Film 4 TE legs	Ag-Modified Bi_0.5_Sb_1.5_Te_3_	60	31.2 mV	1.4 × 10^3^ μW/cm^2^	Flexible	Flexible/wearable substrates	[23]
Double elastomer layer design 12 × 12 array	TE alloys	10	-	25.1 μW/cm^2^	Flexible, Stretchable	Wearables	[93]
TEG module 6–18 pairs of n-type and p-type module	Bi_2_Te_3_ legs with nickel coating, aluminum nitride, gold-coated copper interconnects	2.5 and 1.2	-	35 μW/cm^2^ and 18 μW/cm^2^	-	Wearables and implantable systems	[65]
Pelletized and FPCB 40 TE elements	Bi_0.5_Sb_1.5_Te_3_ and Bi_2_Te_2.7_Se_0.3_, Ni coating, Au coating	1.43	12.1 mV	6.97 μW/cm^2^	Flexible	Wearables and implantable systems	[16]
Paper-based TEGs	Bi_0.5_Sb_1.5_Te_3_ and Bi_2_Te_2.7_Se_0.3_, Paper	35	8.0 mV	0.530 × 10^−3^ μW/cm^2^	Flexible, Stretchable, Eco-friendly	Wearables	[94]
Film-based WTEG	SWCNTs	6	71 mV	5.517 × 10^−3^ μW/cm^2^	Flexible	Wearables	[17]
Film-based TEG	Bi_2_Te_3_	24	48.9 mV	0.693 μW (no area information)	Flexible	Wearables	[15]
CNT-based TENG	SWCNTS and PMDS	50	23 mV	0.166 μW/cm^2^	Flexible, Stretchable, Eco-friendly	Wearables	[95]
Hybrid	Bi_2_Te_3_—SWCNTs	25	17 mV	930 μW/cm^2^	Flexible	Wearables	[22]
Hybrid	PEDOT:PSS/TiS2	30	534 mV	47.8 µW/cm^2^	Flexible	Flexible systems	[20]

**Table 4:** The power consumption of the microcontroller unit with BLE.

Operational Modes	Power Requirement of MCU–BLE		
	TI CC2650 [108]	STM32L476RG [109]	PSoC™ 4 BLE [110]
V_BAT_, supply for RTC and 32X32-bit backup registers		513–1080 nW	-
Shutdown mode (5 wakeup pins)	170–380 nW	51.3–108 nW	285–825 nW
Standby mode (5 wakeup pins)		205.2–432 nW	-
Standby mode with RTC	1.7–3.8 μW	718.2–1512 nW	2.47–6.5 μW
Stop mode		1.881–3.96 μW	-
Stop with RTC		2.394–5.04 μW	114–300 nW
Run mode	10.03–22.42 mW (RX)	171–360 μW/MHz (LDO mode)	-
Run mode	103.7–231.8 μW/MHz	66.69–140.4 μW/MHz (@3.3 v SMPS mode)	-

## Data Availability

No new data were created or analyzed in this study.
